# Lymphotoxin β receptor-mediated NFκB signaling promotes glial lineage differentiation and inhibits neuronal lineage differentiation in mouse brain neural stem/progenitor cells

**DOI:** 10.1186/s12974-018-1074-z

**Published:** 2018-02-20

**Authors:** Xiao Xiao, Raj Putatunda, Yonggang Zhang, Priya V. Soni, Fang Li, Ting Zhang, Mingyang Xin, Jin Jun Luo, John R. Bethea, Yuan Cheng, Wenhui Hu

**Affiliations:** 1 Department of Neurosurgery, The Second Affiliated Hospital of Chongqing Medical University, Chongqing, 400010 China; 2 Center for Metabolic Disease Research, Department of Pathology and Laboratory Medicine, Temple University Lewis Katz School of Medicine, 3500 N Broad Street, Philadelphia, PA 19140 USA; 3 Department of Neurology, Temple University Lewis Katz School of Medicine, 3401 N Broad Street, Philadelphia, PA USA; 4 Department of Biology, Drexel University, Philadelphia, PA USA

**Keywords:** Lymphotoxin, Neural stem cells, NFκB, Neural differentiation, Transgenic mice

## Abstract

**Background:**

Lymphotoxin (LT) is a lymphokine mainly expressed in lymphocytes. LTα binds one or two membrane-associated LTβ to form LTα_2_β_1_ or LTα_1_β_2_ heterotrimers. The predominant LTα_1_β_2_ binds to LTβ receptor (LTβR) primarily expressed in epithelial and stromal cells. Most studies on LTβR signaling have focused on the organization, development, and maintenance of lymphoid tissues. However, the roles of LTβR signaling in the nervous system, particularly in neurogenesis, remain unknown. Here, we investigated the role of LTβR-mediated NFκB signaling in regulating neural lineage differentiation.

**Methods:**

The C57BL/6J wild-type and GFAP-dnIκBα transgenic mice were used. Serum-free embryoid bodies were cultured from mouse embryonic stem cells and further induced into neural stem/progenitor cells (NSCs/NPCs). Primary neurospheres were cultured from embryonic and adult mouse brains followed by monolayer culture for amplification/passage. NFκB activation was determined by adenovirus-mediated NFκB-*firefly*-luciferase reporter assay and p65/RelB/p52 nuclear translocation assay. LTβR mRNA expression was evaluated by quantitative RT-PCR and LTβR protein expression was determined by immunohistochemistry and Western blot analysis. Multilabeled immunocytochemistry or immunohistochemistry followed by fluorescent confocal microscopy and quantitative analysis of neural lineage differentiation were performed. Graphing and statistical analysis were performed with GraphPad Prism software.

**Results:**

In cultured NSCs/NPCs, LTα_1_β_2_ stimulation induced an activation of classical and non-classical NFκB signaling. The expression of LTβR-like immunoreactivity in GFAP^+^/Sox2^+^ NSCs was identified in well-established neurogenic zones of adult mouse brain. Quantitative RT-PCR and Western blot analysis validated the expression of LTβR in cultured NSCs/NPCs and brain neurogenic regions. LTβR expression was significantly increased during neural induction. LTα_1_β_2_ stimulation in cultured NSCs/NPCs promoted astroglial and oligodendrocytic lineage differentiation, but inhibited neuronal lineage differentiation. Astroglial NFκB inactivation in GFAP-dnIκBα transgenic mice rescued LTβR-mediated abnormal phenotypes of cultured NSCs/NPCs.

**Conclusion:**

This study provides the first evidence for the expression and function of LTβR signaling in NSCs/NPCs. Activation of LTβR signaling promotes glial lineage differentiation. Our results suggest that neurogenesis is regulated by the adaptive immunity and inflammatory responses.

**Electronic supplementary material:**

The online version of this article (10.1186/s12974-018-1074-z) contains supplementary material, which is available to authorized users.

## Background

Neurogenesis involves the proliferation, migration, and lineage differentiation of neural stem/progenitor cells (NSCs/NPCs) during development and adult life. Defects or impairments in embryonic and adult neurogenesis have been implicated in a large array of neurodevelopmental disorders [1–6] and neurodegenerative diseases [7–10]. Optimizing adult neurogenesis to achieve functional recovery in patients with various types of nervous system injuries and diseases remains a daunting challenge. NSCs are characterized by their self-renewal and multipotent differentiation into various neural cell lineages, which depend upon the orchestral interplay between the intrinsic cellular properties and extrinsic environmental factors including immunity-related cytokines and chemokines [11, 12]. Neuroinflammatory responses exhibit a double-edged sword role in neurogenesis [13, 14] and neurodegeneration [15, 16].

The nuclear factor κB (NFκB) is well known for its critical role in inflammation, immunity, cancer, and neural plasticity. In most cell types, NFκB maintains cellular growth (proliferation) and survival. However, little is known about the role of NFκB signaling in regulating neural differentiation of NSCs. Activation of NFκB by Toll-like receptor 2 (TLR2) induces neuronal differentiation whereas TLR4 activation inhibits neuronal differentiation of NSCs [17]. In p50 knockout mice, adult hippocampal NSCs exhibit 50% reduction in neuronal differentiation while their proliferative capability does not change [18]. Our previous study showed that selective inhibition of the classical NFκB signaling retains the tripotent ability of differentiation and promotes self-renewal capability of NSCs, suggesting a role of NFκB signaling in mediating the differentiation of NSCs into NPCs at the very early stage [19].

Inflammatory mediators such as cytokines and chemokines have been shown to regulate neurogenesis [20–23]. Tumor necrosis factor (TNF) α has been extensively studied but its role in modulating neurogenesis remains controversial. The enhancing effect of TNFα on neuronal differentiation was first identified in rat NSCs [24]. TNFα promotes neurosphere aggregation in vitro [25]. TNF receptor 2(TNFR2)-derived peptide promotes neuronal lineage differentiation of NSCs [26]. However, TNFα treatment has no effect on neuronal differentiation and neurite outgrowth of murine adult NSCs [27, 28] and mouse embryonic stem cell-derived NPCs [29]. Several studies have demonstrated that TNFα promotes glial, rather than neuronal, lineage differentiation in NSCs [29–32]. Increasing evidence shows that some cytokines, such as IL-1β and IL-6, inhibit neuronal differentiation but promote glial lineage differentiation both in vitro and in vivo [29, 32–42] while others in contrast, such as interferon-γ, inhibit the proliferation but promote neuronal differentiation of NSCs [28, 29, 32, 43, 44]. Noteworthy, different outcomes induced by these factors reflect a different intricate mechanism in regulation of neurogenesis and imply that other inflammatory mediators may also share similar intracellular signal pathways to regulate neurogenesis.

Lymphotoxin (LT) is a lymphokine mainly expressed in T and B lymphocytes. LTα (also known as TNFβ) is devoid of a transmembrane domain and self-associates to form homotrimers LTα_3_ that binds to TNFR [45]. LTα also binds to one or two membrane-associated LTβ to form LTα_2_β_1_ and LTα_1_β_2_ heterotrimers, respectively [46, 47]. The predominant heterotrimer LTα_1_β_2_ binds to LTβ receptor (LTβR) primarily expressed on epithelial and stromal cells [48] and cells of the myeloid lineage [49]. Most studies on LTβR signaling have focused on the organization, development, and maintenance of lymphoid tissues [48] and their role in adaptive and innate immune responses [50]. LTβR signaling modulates follicular dendritic cell maintenance and germinal center formation in lymph nodes [51]. Soluble LTβR-immunoglobulin fusion protein treatment disrupts follicles, marginal zone and germinal center in spleen [52]. LTβR signaling contributes to the formation of tertiary lymph organ, the unique immune cell aggregates at the sites of persistent inflammation [53–55].

However, the roles of LTβR signaling in the nervous system, particularly during neurogenesis, have not yet been investigated. The aims of this study were to determine whether LTβR is expressed in NSCs/NPCs both in vitro and in vivo and to identify the role of LTβR-mediated classical and non-classical NFκB pathways in regulating neural lineage differentiation of NSCs/NPCs.

## Methods

### Reagents and antibodies

TNFα, LTα_1_β_2_, IL-1β, B-cell activating factor (BAFF), CD40L, and LIGHT were obtained from PeproTech. APQ [NF-κB activation inhibitor, 6-amino-4-(4-phenoxyphenylethylamino)quinazoline] was from EMD Chemicals and dissolved in DMSO. Antibodies against LTβR(M-110), Calretinin(N-18), Doublecortin (DCX, C-18), Myelin basic protein (MBP, D-18), Sox2(Y-17), p65(C-20), GAPDH and Lamin A/C(636) were obtained from Santa Cruz Biotechnology. RelB and p100/p52 were from Cell Signal Technology. Mouse anti-NeuN antibody was from Millipore. Chicken anti-glial fibrillary acidic protein (GFAP), anti-Neuron-specific class III β-tubulin (Tuj1), and anti-Nestin antibodies were from Aves. Rabbit anti-GFAP (Z0334) was from Dako. All other reagents were from Sigma.

### Animals and brain specimen collection

All procedures for animal usage were complied with the guidelines of the National Institutes of Health and approved by the Institutional Animal Care and Use Committee (IACUC) at Temple University. The transgenic (TG) mice carrying the NFκB super-inhibitor dominant-negative IκBα (dnIκBα) transgene driven by the promoter of Glial fibrillary acidic protein (GFAP) were obtained from Dr. Bethea’s Lab at the University of Miami and maintained by breeding heterozygous GFAP-dnIκBα males with wild-type (WT) females [56]. WT littermates were used as controls. After euthanization, the brains were collected and different regions of interest were dissected for further studies. Samples for RNA and protein extractions were snap-frozen in liquid nitrogen and stored at − 80 °C.

### Neurosphere and monolayer cultures of NSCs/NPCs

Primary neurospheres were cultured from the subventricular zone (SVZ) of adult mice (2–3 months old, *n* = 3) as described previously [19, 57]. Briefly, whole brains were cut into 0.5-mm-thick coronal slices using mouse stainless steel brain matrices. The SVZ tissues were dissected out carefully and cut into small pieces, digested with Accutase (Sigma, St. Louis, MO) for 25–30 min, and triturated. The dissociated cells were cultured at a density of 2 × 10^5^ cells/ml in DMEM/F12 NSC/NPC proliferation medium containing × 1 B27 Supplement, 20 ng/ml epidermal growth factor (EGF), 10 ng/ml basic fibroblast growth factor (bFGF), and 0.2% Heparin (StemCell Technologies). Medium was changed every other day, and primary neurospheres were formed within 3–7 days.

For expansion, the adherent monolayer culture was performed by dissociating the primary neurospheres with Accutase and seeding in plates coated with matrigel. Half volume of the proliferation medium was changed every other day. Neural lineage differentiation of the monolayer culture was initiated by replacing the proliferation media with the differentiation media containing DMEM/F12, × 1 B27 and × 1 N2 Supplement without EGF/bFGF.

### Neural induction of mouse embryonic stem cells (ESCs)

Mouse ESCs from Transgenic Core Facility at Temple University were cultured in ESC media containing Advanced DMEM (Gibco 12,491), 10% fetal bovine serum (FBS), × 1 Penicillin/Streptomycin/Glutamine (Invitrogen), and 0.1 mM 2-Mercaptoethanol. After initial culture on 60-mm dish for 2–4 h to allow the attachment of mouse embryonic fibroblast, the suspension media containing ESCs were transferred to tissue culture 6-well plate, and cultured at 37 °C, 5% CO2 with daily media change until ESC colonies were formed. Serum-free EB was generated following previously described protocol [58]. Briefly, ESC colonies were harvested without dissociation and re-plated on suspension 6-well plate for 1–3 days to generate EB. Then, the EBs were re-plated in neural induction medium containing equal volume of DMEM/F12 with N2 supplement (× 1) and Neurobasal Medium with B27 supplement (× 1), plus × 1 Penicillin/Streptomycin/Glutamine. After 7 days, serum-free, floating EB had developed. Further culture for 7–14 days in NSC/NPC proliferation medium as described above generated NSCs/NPCs.

### Adenovirus-mediated NFκB-*firefly*-luciferase reporter assay

NSCs/NPCs were cultured in a 96-well plate and infected with adenovirus carrying the NFκB-*firefly*-luciferase reporter (Vector Biolabs) at a multiplicity of infection of 10 for 24 h. The cells in 6 wells per group were treated with or without indicated cytokines for 24 h, including TNFα (10 ng/ml), IL1β (10 ng/ml), LTα_1_β_2_ (100 ng/ml), BAFF (100 ng/ml), CD40L (100 ng/ml), and LIGHT (100 ng/ml). The cell lysate was prepared for measuring of *firefly*-luciferase activity using the ONE-Glo luciferase assay system (Promega). Luminescence was quantified in a 2104 EnVision® Multilabel Reader (PerkinElmer).

### Quantitative reverse transcription-polymerase chain reaction (RT-qPCR)

Cultured ESCs, EBs, NSCs/NPCs, or snap-frozen brain tissues were processed with an RNeasy Mini kit (Qiagen) according to the manufacturer’s instruction for total RNA extraction. RNase-Free DNase Set (Qiagen) was used to remove any potentially residual genomic DNA through on-column DNase digestion. High Capacity cDNA Reverse Transcription Kit (Invitrogen, Grand Island, NY) was used for cDNA synthesis from 2 μg of RNA for each sample using random hexanucleotide primer. Quantitative PCR (qPCR) was performed in a BioRad CFX qPCR instrument using an SYBR® Green PCR Master Mix Kit (BioRad). One pair of mouse LTβR primers targeted the range 786–923 nucleotide (5′-gcagctccaggtacctcctactcg-3′ on exon-6 and 5′-cctcatccaggcacaggccaggac-3′ on exon-7) of mouse LTβR (NM_020736.3). The primers for mouse housekeeping gene Ppia were forward 5′-gcccagtatgcttgggtatc-3′ and reverse 5′-tgctgactcccagaacaga-3′, or β-actin were forward 5′-aagagctatgagctgcctga-3′ and reverse 5′-tacggatgtcaacgtcacac-3′. Each sample was tested in triplicate. Cycle threshold (Ct) values were obtained graphically for LTβR and Ppa2 or β-actin. Their difference (ΔCt) was used to obtain ΔΔCt values by subtracting the ΔCt values of control samples from those of experimental samples. Relative fold change in gene expression was calculated as 2-ΔΔCt.

### Western blot analysis

Culture NSCs/NPCs or snap-frozen brain tissues were lysed at 4 °C for 30 min in Triton X-100-based lysis buffer (20 mM Tris-HCl (pH 7.4), 1% Triton X-100, 5 mM ethylenediaminetetraacetic acid, 5 mM dithiothreitol, 150 mM NaCl, 1 mM phenylmethylsulfonyl fluoride, × 1 proteinase inhibitor cocktail (Cayman Chemical, Ann Arbor, MI), 1 mM sodium orthovanadate and 30 mM NaF). After centrifugation at 20,000*g* for 20 min at 4 °C, the supernatant was collected for protein concentration determination with a Pierce BCA Protein Assay Kit (cat# 23225). An equal amount of protein lysate (20 μg) was resolved by the SDS-polyacrylamide gel electrophoresis system and transferred to nitrocellulose membrane (BioRad). The SeeBlue prestained protein standards (Invitrogen) were used as a molecular weight reference. The Odyssey CLx Infrared Fluorescent Western Blot system (LI-COR, Lincoln, NE) was used according to the manufacture’s instruction. Briefly, after blocking with Odyssey blocking buffer containing 0.1% (*v*/*v*) Tween 20, the membranes were incubated overnight at 4 °C with goat anti-LTβR polyclonal antibody (1:500, SC-8376, Santa Cruz) and mouse anti-GAPDH monoclonal antibody (1:1000, Sigma). After washing three times, the membrane was incubated with fluorescently conjugated secondary antibodies (IRDye 680LT-conjugated anti-mouse or IRDye 800CW-conjugated anti-rabbit) for 1 h at room temperature. The membranes were scanned and analyzed using the Odyssey Infrared Imaging System. Relative signal intensities were determined using the LI-COR imaging software, which is independent of the image intensity adjustment.

### Immunocytochemical staining of cultured NSCs/NPCs and immunohistochemical staining of mouse brain tissues

Cultured NSCs/NPCs upon differentiation in 16-well chambered slides were fixed for 20 min in 2% paraformaldehyde and 25% sucrose in PBS. After three rinses, the cells were treated with 0.5% Triton X-100 PBS for 20 min and blocked by 10% donkey serum or 2% bovine serum albumin/PBS for 2 h. Cells were incubated overnight at 4 °C with indicated primary antibodies. After washing three times, cells were incubated with corresponding secondary antibodies for 1 h and with DAPI for 5 min. After three rinses with PBS, the cells were mounted with a coverslip using antifading aqueous mounting media (Biomeda, Foster City, CA) and analyzed under fluorescence microscope and confocal imaging analysis.

For multilabeled immunohistochemistry, mice were euthanized with an overdose of pentobarbital solution and transcardially perfused with 4% paraformaldehyde. The brains were dissected, postfixed overnight in the same fixative, and cryopreserved with buffered 30% sucrose. A series of coronal sections of brain at 40 μm thickness were cut on a freezing sliding microtome and collected in a serial manner and stored at − 20°C. Then, standard multiple-labeled immunofluorescent staining was performed. Briefly, sections were permeated for 30 min in PBS containing 0.5% Triton X-100 and blocked with 10% normal donkey serum for 30 min. The sections were incubated with indicated primary antibodies in PBS with 0.1% Triton X-100 overnight at 4 °C. After washing three times each for 10 min, the sections were incubated with corresponding Alexa Fluor® conjugated donkey secondary antibody (1:400; Invitrogen, Grand Island, NY) for 1 h at room temperature. DAPI was used for nuclear counterstaining. After three rinses with PBS, sections were mounted on a slide and coverslipped with antifading aqueous mounting media (Biomeda, Foster City, CA).

For confocal imaging analysis, samples were viewed on a Leica SP8 confocal microscope system. Differential visualization of three or four fluorophores Alexa-488, Alexa-594, Alexa-680 and DAPI was obtained via specific filter combinations. Samples were scanned sequentially to avoid any potential for fluorophore bleed-through. The Z-stack scanning images (1024 × 1024 pixels) through 0.5 or 1.0 μm optical sections were obtained under identical exposure conditions and processed through the build-in processing tools. For quantification analysis, at least 8 images were collected for each condition and the Z-stack images for equal number of Z-stacks were maximized for further identification and manual counting of indicated positive cells. The percentage of indicated positive cells over total DAPI-positive nucleus was calculated.

### Statistical analysis

All statistical analysis was performed using Prism GraphPad 6.0. Significance was determined between multiple genotypes using a one-way ANOVA with Tukey post-hoc analysis. An unpaired two-tailed Student’s *t* test was performed between two groups of different treatments. The *p* value at < 0.05 and < 0.01 were used for statistical significance.

## Result

### Lymphotoxin α_1_β_2_ (LTα_1_β_2_) activates classical and non-classical NFκB signaling pathways in neural stem/progenitor cells

**Fig. 1:**
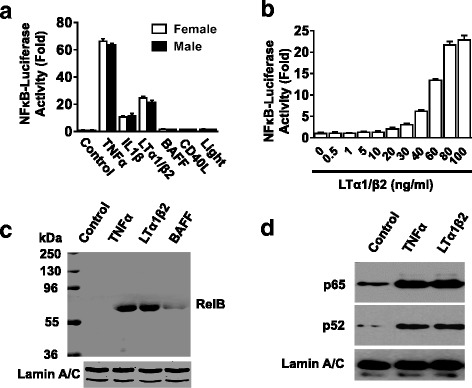
Ltα1/β2 activates classical and non-classical NFκB signaling pathway in mouse neural stem/progenitor cells (NSCs/NPCs). **a**, **b** Adenovirus-mediated NFκB-*firefly*-luciferase reporter assay showing a comparison of classical and non-classical NFκB stimulators (**a**) and a dose response of Ltα1/β2 (**b**) in NSCs/NPCs. Dissociated NSCs/NPCs cultured from adult mouse brain subventricular zones (SVZ) were plated on 96-well plate and infected with adenovirus carrying NFκB *firefly*-luciferase at 50 multiplicity of infection for 24 h and treated with indicated cytokines for 24 h. Luciferase activity was measured using OneGlo luciferase kit. Data are expressed as relative fold changes compared with corresponding control. **c**, **d** Western blot analysis of nuclear extracts from SVZ NSCs/NPCs for the nuclear translocation of non-classical (RelB, p52) and classical (p65) NFκB pathways. NSCs/NPCs were treated with indicated cytokines for 4 h before preparation of nuclear extracts. TNF: tumor necrosis factor; LT: lymphotoxin; IL: interleukin. Lamin A/C served as nuclear protein loading control

In our previous study, we found that LTα_1_β_2_ stimulates activation of NFκB-luciferase reporter in mouse embryonic/neonatal SVZ NSCs/NPCs and enteric neuronal cell line [19], indicating the immunological impact on the neurogenesis in the developmental period [59]. To corroborate this observation in adult neurogenesis, we repeated similar NFκB-luciferase reporter assay in SVZ NSCs/NPCs from adult mice (2–3 months old). We examined various stimulators for classical and non-classical NFκB signaling pathways [19, 60]. Although the three selected cytokines TNFα and IL-1β (the best-known activators for the classical NFκB pathways) as well as LTα_1_β_2_ (for both pathway) [61–66] induced significant activation of NFκB-luciferase reporter in adult SVZ NSCs/NPCs, the induction pattern in adult NSCs/NPCs exhibited slight difference from embryonic NSCs/NPCs [19], with lower induction by LTα_1_β_2_ v.s. TNFα in adult SVZ NSCs/NPCs (Fig. 1a). Interestingly, similar induction patterns occurred in both male and female littermate mice (Fig. 1a); thus, both genders were used randomly in the following studies. The LTα_1_β_2_-induced NFκB activation was dose-dependent with a narrow window (Fig. 1b). However, the selected cytokines BAFF and CD40L (well-known activators for the non-classical pathway) and LIGHT (for both pathway) [67–69] had no effects on NFκB-luciferase reporter activity in cultured adult SVZ NSCs/NPCs (Fig. 1a), consistent with our previous observation on BAFF in embryonic SVZ NSCs/NPCs [19]. The response to BAFF signaling was confirmed by Western blotting showing the nuclear translocation of RelB, a main marker for non-classical pathway (Fig. 1c). However, LTα_1_β_2_ treatment induced the nuclear translocation of RelB and p52 for non-classical and p65 for classical pathway in adult NSCs/NPCs (Fig. 1c, d), which is also consistent with our previous observation in mouse enteric neuronal cell line [19]. Taken together, administration of LTα_1_β_2_ induces activation of both classical and non-classical NFκB pathways in mouse SVZ NSCs/NPCs.

### LTβR expression exists in neural stem cells both in vitro and in vivo

**Fig. 2:**
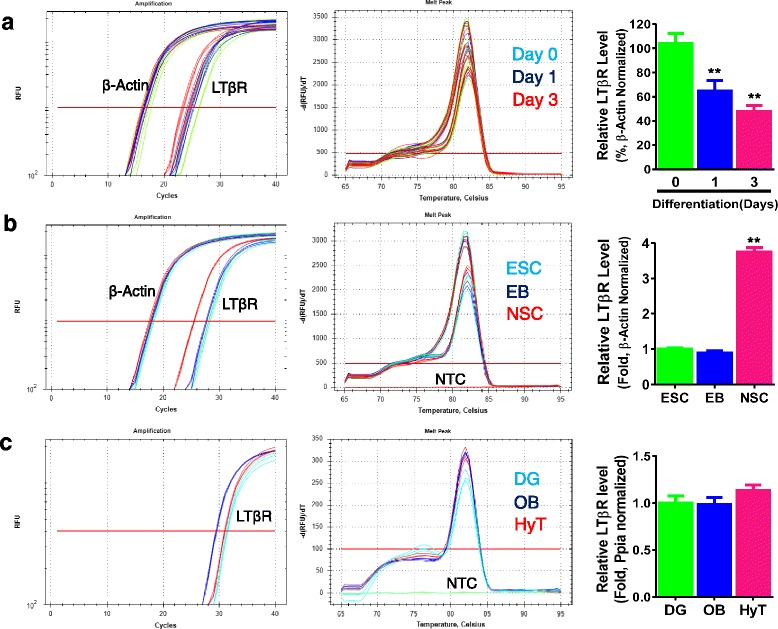
LTβR mRNA expression is enriched in mouse neural stem/progenitor cells. **a** Relative levels of LTβR mRNA expression in adult mouse SVZ NSCs/NPCs under proliferation or differentiation for 1 and 3 days. **b** Significant increase in LTβR mRNA levels during neural induction from embryonic stem cells (ESC), embryoid body (EB) to NSC. **c** Presence of LTβR mRNA expression in neurogenic tissues of adult mouse brain. In all the relative RT-qPCR analysis, representative amplification curve (log scale) for cycle threshold (Ct) values and melt peak curve for validation of pure PCR product are presented. Relative quantification of LTβR PCR product crossing exon 6–7 after β-actin or Ppia normalization. Data represent mean ± SEM of 3 experiments. **p < 0.01 indicate significant changes as compared to corresponding NSC (**a**) or ESC (**b**) group

**Fig. 3:**
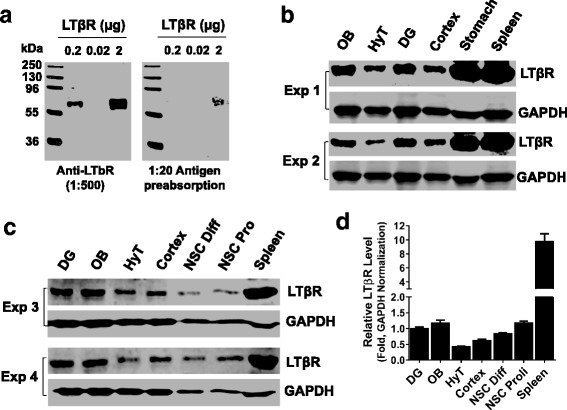
Western blot analysis validates the presence of LTβR protein expression in adult mouse SVZ NSCs/NPCs. **a** Specificity of anti-LTβR antibody. **b**, **c** Western blot analysis showing relative levels of LTβR protein expression in indicated brain regions. The spleen tissue was used as a positive control. **d** Bar graph showing the densitometry data representing mean ± SEM of 4 independent experiments with relative fold changes as compared with the level in DG after GAPDH normalization. DG, dentate gyrus; OB, olfactory bulb; HyT, hypothalamus; Pro, proliferation; Diff, differentiation

**Fig. 4:**
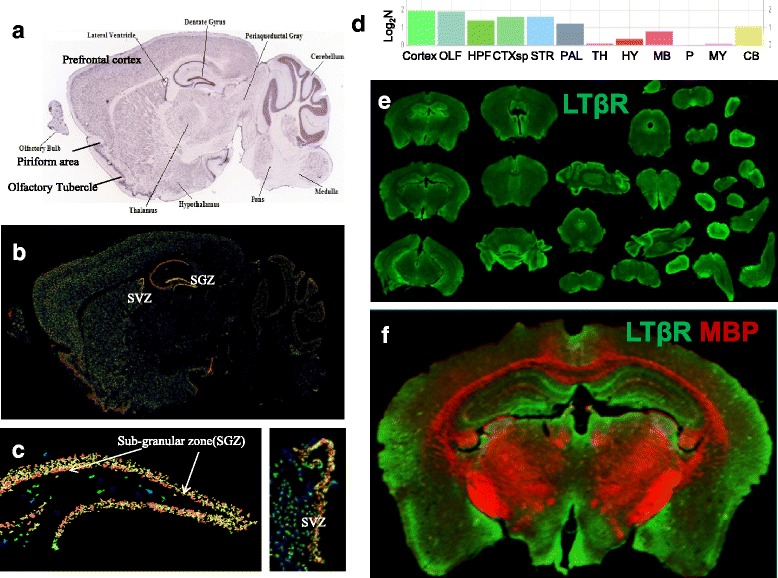
Extensive expression of LTβR mRNA (**a**–**d**) and protein (**e**, **f**) in mouse brain, primarily on neurogenic zones (SGZ, SVZ), prefrontal cortex, hypothalamus and cerebellum. **a**–**c** In situ hybridization data of adult mouse brain sagittal section using LTβR antisense probe was collected from http://mouse.brain-map.org/gene/show/16770. Representative merged view of dentate gyrus and SVZ (**c**) showed strong expression of LTβR mRNA around the SGZ, granular layer and SVZ. **d** Relative expression levels of raw expression values (Log_2_:N). **e**, **f** Series coronal sections of adult mouse brain were immunostained for LTβR (green, 800 nm channel) and MBP (red, 700 nm channel) and scanned with Licor Odyssey CLX imager, showing expression pattern similar to in situ hybridization

**Fig. 5:**
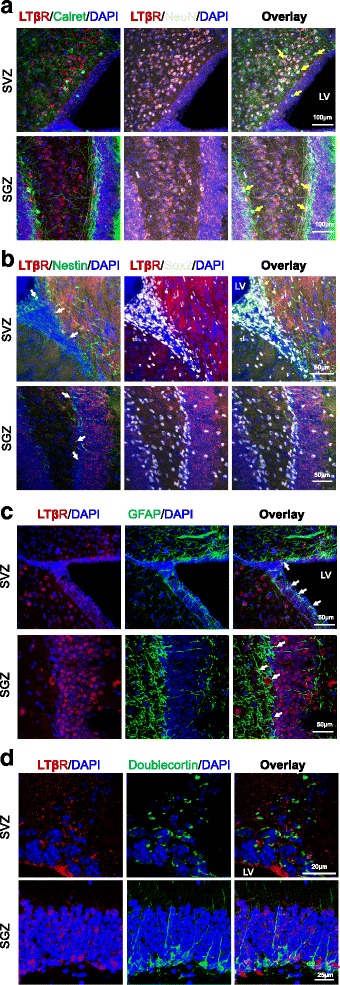
Expression of LTβR-like immunoreactivity in subventricular zone (SVZ) and subgranular zone (SGZ) of adult mouse brain. Coronal brain sections underwent free-floating immunofluorescent immunohistochemistry with rabbit anti-LTβR antibody (red) together with goat anti-Calretinin (green) and mouse anti-NeuN (white) antibodies (**a**), chicken anti-Nestin (green) and goat anti-Sox2 (white) antibodies (**b**), chicken anti-GFAP (green) antibody (**c**), or goat anti-DCX (green) antibody (**d**) followed by corresponding Alexa Fluor® conjugated secondary antibodies and confocal image analysis. LTβR is primarily expressed in GFAP^+^/Sox2^+^ NSC (white arrow), some neuroblasts (red arrow) and mature neurons (yellow arrow). LV, lateral ventricle; DG, dentate gyrus

Previous studies have shown that LTβ receptor (LTβR) is expressed primarily in epithelial and stromal cells [49, 70]. There are no conclusive reports about the expression of LTβR in brain and neural cells. Our observation on the LTα_1_β_2_-induced activation of NFκB signaling in NSCs/NPCs indicates the presence of its receptor LTβR in these cells. To validate this, we first determined whether LTβR mRNA was expressed in cultured NSCs/NPCs from adult mouse SVZ. The melting peak curve of RT-qPCR reaction attested to the presence of LTβR mRNA expression in cultured NSCs/NPCs (Fig. 2a), which was significantly reduced after NSC/NPC differentiation for 1–3 days (Fig. 2a). Our previous study showed the NFκB reporter response of embryonic NSCs/NPCs to LTα1β2 [19], implying the presence of LTβR expression during embryonic neurogenesis. To corroborate this and explore the potential role of LTβR-mediated immune regulation during neural induction, we examined the expression pattern of LTβR mRNA in ES cells, EB, and NSCs. We found that both ES cells and EB do express LTβR mRNA as evidenced by the melting peak curve, but the expression was much lower than that in NSCs (Fig. 2b). The presence of LTβR mRNA expression during adult neurogenesis was also validated by RT-qPCR analysis using the well-established neurogenic zones of adult mouse brains (Fig. 2c). To determine the presence of LTβR protein expression in NSCs/NPCs and brain tissues, we performed Western blot analysis using anti-LTβR antibody, which specificity was verified using recombinant LTβR protein (from AbCam, ab198752) and 1:20 antigen preabsorption (Fig. 3a). When using spleen tissue as a positive control, the protein expression of LTβR was detected in adult brain tissues, with relatively higher levels in neurogenic regions such as dentate gyrus (DG) and olfactory bulb (OB) than the rest regions tested in the brain but significantly lower than that in the spleen tissue (Fig. 3b–d). LTβR protein expression was also detectable in NSCs/NPCs cultured under proliferation or differentiation conditions (Fig. 3c, d). To address the regional and cellular distribution of LTβR expression in the adult brain, we first analyzed the public in situ hybridization data in the sagittal brain sections of 8-week-old male C57/BL/6J mouse (http://mouse.brain-map.org) using LTβR antisense probe generated by PCR with primer forward 5′-cctcctactcggataccatctg-3′ and reverse 5′-atcctagtgtctctgtctcggc-3′. LTβR mRNA was extensively expressed in the mouse brain (Fig. 4a–d), predominantly in the neurogenic zones (SGZ, SVZ), prefrontal cortex, hypothalamus, and cerebellum. In particular, the granular cells in DG showed very high expression (Fig. 4c). Then, we performed immunohistochemistry with anti-LTβR antibody using series coronal sections of adult mouse brains. Anti-MBP antibody was used to demarcate the white matter. The pattern of the regional distribution of LTβR-like immunoreactivity was similar to that of mRNA expression, namely predominant in the neurogenic zones such as SVZ, SGZ, hypothalamic arcuate nucleus, and amygdala (Fig. 4e, f). To validate the expression of LTβR protein in NSCs/NPCs of mouse neurogenic zones, we performed multilabeled immunofluorescent staining and confocal image analysis. We observed that LTβR-like immunoreactivity existed mainly in NeuN/Calretinin-positive neurons (Fig. 5a) and Sox2/Nestin/GFAP-positive NSCs (Fig. 5b, c), but weakly expressed in Sox2/Nestin-positive NPCs (Fig. 5c) and DCX-positive neuroblasts (Fig. 5d). These data suggest that LTβR is expressed at both mRNA and protein levels in NSCs both in vitro and in vivo.

### LTα_1_β_2_ inhibits neuronal differentiation and promotes glial lineage differentiation of NSCs/NPCs

**Fig. 6:**
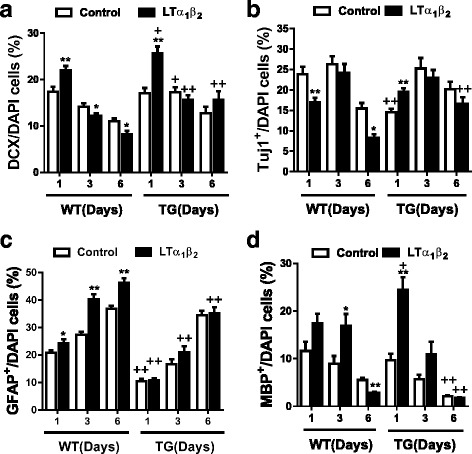
Transgenic inactivation of astroglial NFκB pathway inhibits constitutive and LT-induced astroglial lineage differentiation but favors neuronal lineage differentiation. **a**, **b** Quantitative analysis of neuronal lineage differentiation determined by Tuj1 (immature neurons) and DCX (neuroblasts). **c**, **d** Quantitative analysis of glial lineage differentiation determined by GFAP (astrocytes) or MBP (Oligodendrocytes). Multi-labeled fluorescent immunocytochemistry and confocal image analysis were performed at 1–6 days of differentiation from SVZ NSCs/NPCs of littermated wild-type (WT) and GFAP-dnIκBα transgenic mice (TG) in the presence or absence of LTα_1_β_2_ (100 ng/ml). The percentage of Tuj1^+^ or DCX^+^ (neurons/neuroblasts), GFAP^+^ (astrocytes) and MBP^+^ (oligodendrocytes) over DAPI-stained cells per field under × 40 objective was determined. Data from 8 fields per duplicate well of each experiment containing littermated WT and TG NSCs/NPCs in 16-well chamberslide from 3 paired animals were statistically analyzed by two-tailed student *t*-test. *p < 0.05 and **p < 0.01 indicate significant changes in LTα_1_β_2_ treatment as compared to corresponding control. ^+^*p* < 0.05 and ^++^*p* < 0.01 indicate significant changes in the TG group as compared with corresponding WT group

Various cytokines exhibit different and frequently opposite effects on neural lineage differentiation of NSCs/NPCs (see the “Background” section). To address whether LTβR signaling affects neural lineage differentiation, we performed multilabeled immunocytochemistry with antibodies against lineage-specific markers Tuj1/DCX, GFAP, and MBP in cultured adult NSCs/NPCs (Fig. 6, Additional file 1: Figure S1a). During differentiation, the proportions of Tuj1-positive neurons and GFAP-positive astrocytes were increased while those of DCX-positive neuroblasts/neuronal cells and MBP-positive oligodendrocytes decreased in a time-dependent manner (Fig. 6). Treatment with LTα_1_β_2_ at the initiation of neural differentiation increased the number at day 1 after treatment of those three lineage neural cells, including DCX-positive neuroblasts/neuronal cells, MBP-positive oligodendrocytes and GFAP-positive astrocytes (Fig. 6) but reduced the number of Tuj1-positive immature neurons (Fig. 6b). However, LTα_1_β_2_ treatment caused diverse responses at the later stage of neural differentiation (day 3–6). It significantly reduced the number of DCX-positive neuroblasts/neuronal cells (Fig. 6a, Additional file 1: Figure S1a) and Tuj1-positive immature neurons (Fig. 6b), but significantly increased the number of GFAP-positive astrocytes (Fig. 6c, Additional file 1: Figure S1a) and MBP-positive oligodendrocytes (Fig. 6d). To further validate the effects of LTα_1_β_2_ on the neural lineage differentiation during embryonic neurogenesis, we treated E14 embryonic NSCs/NPCs with LTα_1_β_2_ in a similar way as that for adult NSCs/NPCs. We observed similar effects on neural lineage differentiation (Additional file 1: Figure S2). Pretreatment with NFκB activation inhibitor APQ dramatically blocked three lineage differentiation with complete loss of both DCX and MBP positive cells (Additional file 1: Figure S3a–c). Upon LTα_1_β_2_ treatment, the increased astroglial lineage differentiation was prevented while neuronal lineage differentiation impairment was aggravated by APQ pretreatment (Additional file 1: Figure S3a–c). These data suggest that LTβR signaling may be involved in the initiation of NSC differentiation into NPCs at the early stage while NFκB inhibition prevents all three lineage differentiation. These findings are consistent with those from our previous studies demonstrating that NFκB signaling is essential to initiate NSC differentiation into NPCs [19] and maintain the late differentiation of three neural lineages [19, 60]. At the later stages of neurogenesis, LTβR-mediated NFκB signaling preferentially stimulates glial lineage differentiation but impairs neuronal lineage differentiation. This is also consistent with other reports showing that TNFα, IL-1β, IL-6 and interferon-γ promote glial lineage differentiation while inhibit neuronal differentiation of NSCs/NPCs [28–44]. Surprisingly, Tuj1 expression was accumulated in the nucleus of most Tuj1-positive neurons after APQ pretreatment regardless whether or not with LTα_1_β_2_ treatment (Additional file 1: Figure S3a).

### Astroglial NFκB inactivation promotes neuronal lineage differentiation and inhibits astroglial lineage differentiation

As shown above, NFκB signaling mediates three lineage differentiation in a stimulus-dependent manner, and cytokines preferentially drive differentiation toward glial lineage cells. To determine if astroglial NFκB signaling mediates LT-induced astroglial lineage differentiation, we used a transgenic (TG) mouse line expressing a dominant-negative IκBα under the GFAP promoter [56]. This mouse line displays a deficit in adult neurogenesis [19] and learning/memory [71]. The specific inhibition of astroglial NFκB signaling was demonstrated in our previous studies [56, 19] and confirmed here. As shown in the Additional file 1: Figure S3d, the activation of NFκB-luciferase reporter with TNFα and LTα_1_β_2_ was significantly suppressed in TG NSCs/NPCs. Astroglial NFκB inactivation suppressed astroglial and oligodendrocytic lineage differentiation in TG NSCs/NPCs as compared with that corresponding to WT NSCs/NPCs at days 1–3 in the absence of LTα_1_β_2_ treatment (Fig. 6c, d). LT-stimulated elevation of astrocytic differentiation was prevented completely by the transgenic inactivation of astroglial NFκB signaling at days 1–6 (Fig. 6c). However, astroglial NFκB inactivation promoted LT-induced oligodendrocytic differentiation at the initial stage (day 1) but inhibited it at later stage (days 3–6) (Fig. 6d). Interestingly, the constitutive neuronal differentiation was significantly improved in TG NSCs/NPCs, and LT-induced inhibition of neuronal differentiation was reversed by the astroglial NFκB inactivation at days 1–6 (Fig. 6a, b). These data suggest that selective inhibition of NFκB signaling in GFAP-positive cells promotes neuronal lineage differentiation while inhibits glial lineage differentiation.

## Discussion

**Fig. 7:**
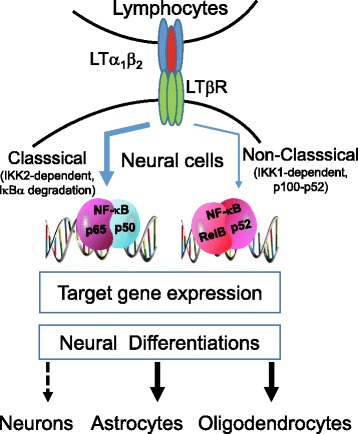
Diagram illustrating the stronger classical and weaker non-classical NFκB signaling pathways induced by LTα_1_β_2_ stimulation in mouse brain neural stem/progenitor cells, resulting in promotion of glial lineage differentiation and inhibition of neuronal lineage differentiation. Selective astroglial inactivation of NFκB signaling inhibits astroglial lineage differentiation while promotes neuronal lineage differentiation

The salient finding in this study is the first identification of LTβR signaling and function in NSCs/NPCs both in vitro and in vivo. Activation of LTβR signaling promotes astroglial and oligodendrocytic, but inhibits neuronal, lineage differentiation. Astroglial inactivation of NFκB signaling compromises astroglial, but promotes neuronal, lineage differentiation (Fig. 7). Our findings suggest that neurogenesis may be deliberately regulated by the adaptive immunity and inflammatory responses via LTβR signaling.

Intensive attention has been paid on inflammatory mediators including cytokines, chemokines, growth factors, and adhesion molecules due to their critical and diverse roles in the pathophysiology related to the disorders of nervous system [21, 22, 72–74]. It is well known that most inflammatory mediators regulate NFκB signaling. NFκB signaling has been widely shown to regulate self-renewal, proliferation, and apoptosis of NSCs/NPCs; migration of neuroblasts; and maturation and plasticity of nascent neurons [73, 75–78]. However, different cytokines may exhibit various and, sometimes, opposite effects on neurogenesis. The current study provides novel evidence to support the critical role of LTβR signaling in regulating neurogenesis. LTβR signaling has long been investigated in lymphoid organogenesis [79], lymphangiogenesis [53–55] and tumorigenesis [80–84]. It involves in many inflammatory processes relative to chronic inflammatory diseases [55] including brain tertiary lymph organ [85]. Although several inflammatory mediators have been shown to regulate neurogenesis [60], no report is seen in the literature up-to-date on the potential role of LT and its receptor signaling in neurogenesis. During our investigation on NFκB signaling in initiating NSC differentiation, we surprisingly found that LTα1β2 induces potent activation of NFκB signaling in embryonic/neonatal NSCs, with similar potency to TNFα [19]. This interesting observation suggests that lymphotoxin-mediated immune responses influences embryonic/neonatal neurogenesis [59, 86]. This also directed us to explore further the effect of LTβR signaling on adult neurogenesis [9, 87]. We demonstrated strong evidence that LTβR signaling is enriched in NSCs during neural induction and neurogenesis. Through in situ hybridization and immunohistochemistry, the presence of extensive expression of LTbR in most neurons were identified, indicating a potential essential role in regulating neuronal plasticity and yet to be determined functions. Our observation also suggests that lymphocyte-derived LT can directly influence NSCs and neurons via its unique receptor LTβR. It is important to understand how the direct communication between lymphocytes and neural cells takes place and how immune system regulates nervous system in both physiological and pathological conditions [14, 59, 86]. The preferential promotion of glial lineage differentiation by LTβR signaling may contribute to neurodegeneration and repair process during nervous system injury and diseases. In multiple sclerosis animal models, LTβR signaling plays a detrimental effect on both demyelination and remyelination via T cells [88, 89] and/or local astrocytes/microglia [90]. Inhibition of LTβR signaling might be a better candidate biotherapeutics for inflammatory demyelinating diseases due to its dual benefits in both delaying demyelination and promoting remyelination [90].

There are three NFκB activation pathways described: classical (canonical), non-classical (non-canonical, alternative), and atypical pathways [91]. LTβR activation engages preferentially the non-classical pathway in immune systems and other cell types [61–66]. Our studies in NSCs/NPCs demonstrate that LTβR preferentially activates the classical pathway, which is supported by the following experimental evidence: (1) LTα_1_β_2_ induced activation of NFκB-luciferase reporter in a similar manner as TNFα and IL-1β; (2) LTα_1_β_2_ dramatically stimulates p65 phosphorylation [84], IκBα degradation [84], and p65 nuclear translocation (Fig. 1d); (3) Transgenic dnIκBα expression in GFAP-positive cells blocked LT-induced astroglial differentiation; (4) LIGHT did not activate NFκB-luciferase reporter although it has widely been shown to bind to LTβR in other cell types [67, 68, 92].

NSC transplantation has been extensively studied in both animal models and clinical trials [93, 94]. A major barrier in the therapeutic application for NSC transplantation is the preferential astrogliogenesis in vivo when NSCs/NPCs encounter the debilitated microenvironmental niches where abundant inflammatory cytokines, including NFκB signaling stimulators, may exist [95–98]. A selective blockade of astrogliogenesis and promotion of neuronal differentiation would be an important strategy for NSC transplantation. Our findings that astroglial NFκB inactivation suppressed LT-induced astrogliogenesis but promoted neuronal lineage differentiation may help approach to establishing a novel therapeutic strategy. Supportively, a previous study using lentivirus-mediated IκBα gene delivery in cultured NSCs/NPCs demonstrated that NFκB inhibition promotes survival and neuronal differentiation of transplanted NSCs [99].

## Conclusions

In conclusion, this is the first report to show LTβR effects on regulating neural differentiation by demonstrating evidence of the expression and function of LTβR signaling in neural stem/progenitor cells. Activation of LTβR signaling promotes lineage differentiation of neural stem/progenitor cells preferentially through classical NFκB pathway. Understanding neuroinflammatory responses and neuroimmunity modulating neurogenesis might help understand the underlying pathophysiology relative to neuro-plasticity and relevant neurological disorders. LTβR may become a potential therapeutic target in the future in treating various neurodevelopmental and neurodegenerative diseases [14, 16].

## Additional file

Additional file 1: **Figure S1.** Representative micrographs showing transgenic inactivation of astroglial NFκB pathway inhibits constitutive and LT-induced astroglial lineage differentiation but favors neuronal lineage differentiation in SVZ NSCs/NPCs from littermated wild-type (WT, a) and GFAP-dnIκBα transgenic mice (TG, b). At the 1st, 3rd and 6th day of differentiation, GFAP (green) and DCX (red)-positive cells were determined by multi-labeled fluorescent immunocytochemistry and confocal image analysis. Scale bars= 50 µm. **Figure S2**. LTα_1_β_2_ promoted astrocytic and oligodendrocytic differentiation but inhibited neuronal differentiation in mouse embryonic NSCs/NPCs. (a, b) Representative micrographs showing 3 lineage differentiation of cultured NSCs/NPCs from E14 mouse brain. White arrows (a) indicate representative Tuj1-positive typical neurons with various degrees of neurites and branches. The small red dots in GFAP staining (b) derived from non-specific debris for chicken anti-GFAP antibody. Scale bars = 75 µm. (c) Quantitative analysis of lineage differentiation after treatment with LTα_1_β_2_. **Figure S3**. Effects of NFκB inhibition on neural lineage differentiation in mouse NSCs/NPCs. (a, b) Representative micrographs showing complete loss of DCX (neuroblasts) and MBP (oligodendrocytes) and dramatic reduction of Tuj1 (immature neurons) and GFAP (astrocytes) after NFκB activation inhibitor APQ (10 µM) pretreatment 30 min before LTα_1_β_2_ (100 ng/ml) treatment under differentiation condition for 3 days. White arrows indicate representative Tuj1-positive typical neurons and green arrows show the nuclear location of Tuj1 expression (a). Scale bars = 75 µm. (c) Quantitative analysis of Tuj1 and GFAP positive cells after LTα_1_β_2_ treatment in the presence or absence of APQ. (d) Adenovirus-mediated NFκB-firefly-luciferase reporter assay showing a significant reduction in cytokine-induced NFκB activation in SVZ NSCs/NPCs from TG mice. Data represent mean ± SEM. * *p*<0.05 and ** *p*<0.01 indicate significant changes after cytokine treatment as compared to corresponding control group. ++ *p*<0.01 indicates significant decrease in APQ group compared with corresponding DMSO group. (PPTX 5201 kb)

## Abbreviations

APQ: 6-Amino-4-(4-phenoxyphenylethylamino)quinazoline
BAFF: B-cell activating factor
bFGF: Basic fibroblast growth factor
DCX: Doublecortin
dnIκBα: Dominant-negative inhibitor of NFκB α
EB: Embryoid body
EGF: Epidermal growth factor
ESC: Embryonic stem cells
GFAP: Glial fibrillary acidic protein
IL-1β: Interleukin-1β
LT: Lymphotoxin
LTβR: Lymphotoxin β receptor
MBP: Myelin basic protein
NFκB: Nuclear factor kappa B
NPC: Neural progenitor cells
NSC: Neural stem cells
PBS: Phosphate-buffered saline
RT-PCR: Reverse transcription-polymerase chain reaction
SGZ: Subgranular zone
SVZ: Subventricular zone
TLR: Toll-like receptor
TNF-α: Tumor necrosis factor-α
Tuj1: Neuron-specific class III β-tubulin
