# Supplementary figures and images for: Lymphotoxin β receptor-mediated NFκB signaling promotes glial lineage differentiation and inhibits neuronal lineage differentiation in mouse brain neural stem/progenitor cells
Source: J Neuroinflammation. 2018 Feb 20;15:49. doi: 10.1186/s12974-018-1074-z (PMC5819232; doi:10.1186/s12974-018-1074-z)

Fig. Sup1

**a** WT: DCX/GFAP/DAPI

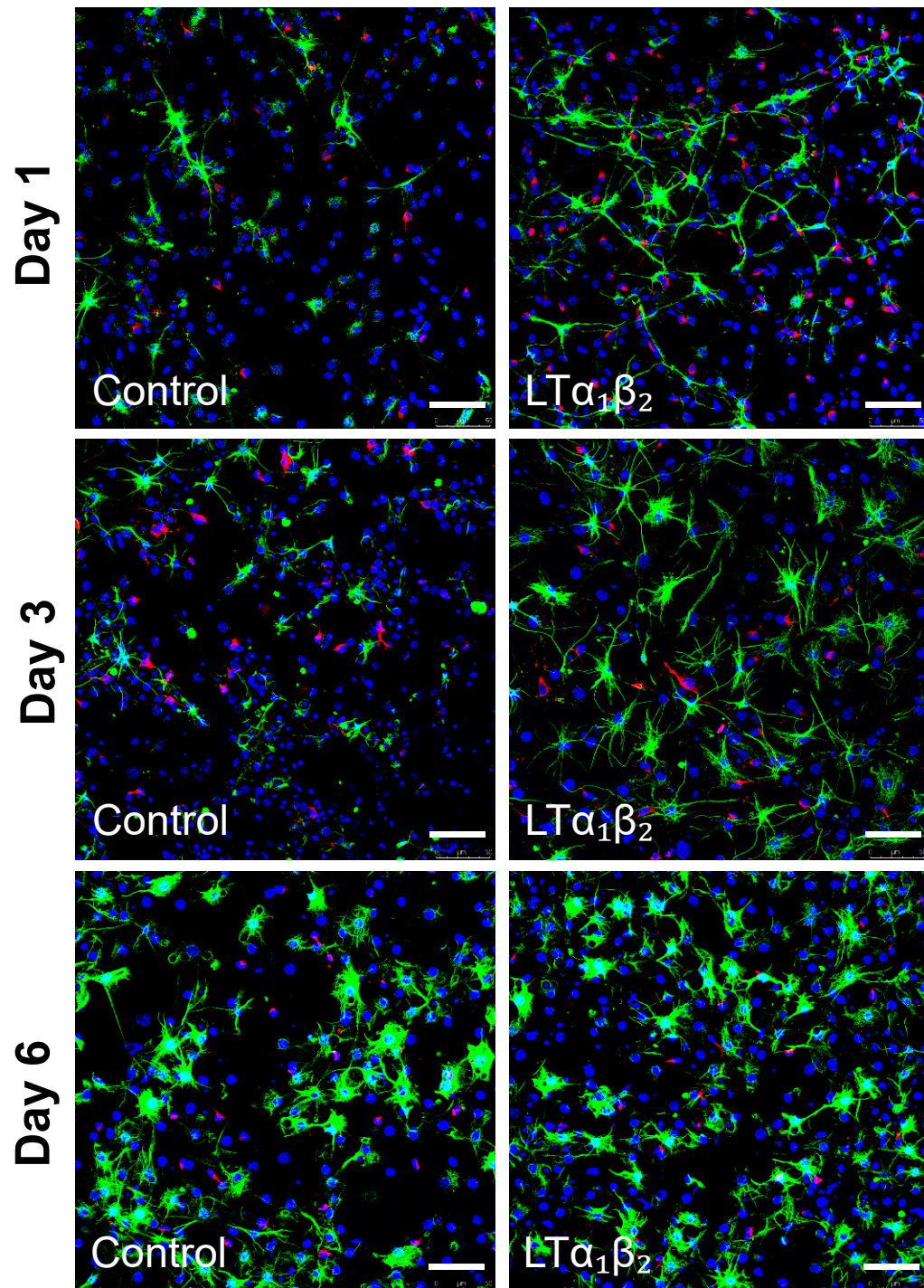

**b** TG: DCX/GFAP/DAPI

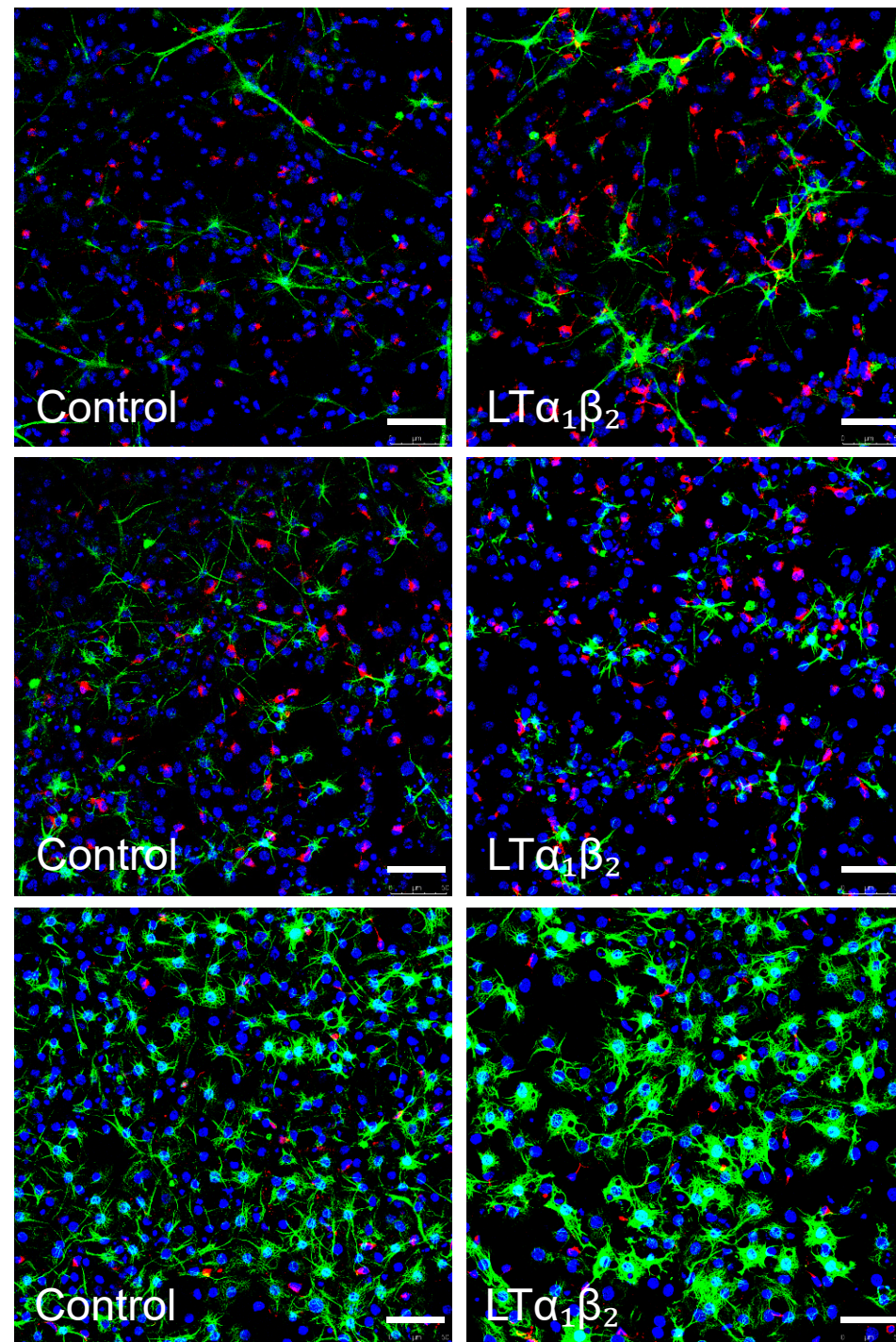

Fig. Sup 2

**a** **Tuj1/MBP/DAPI**

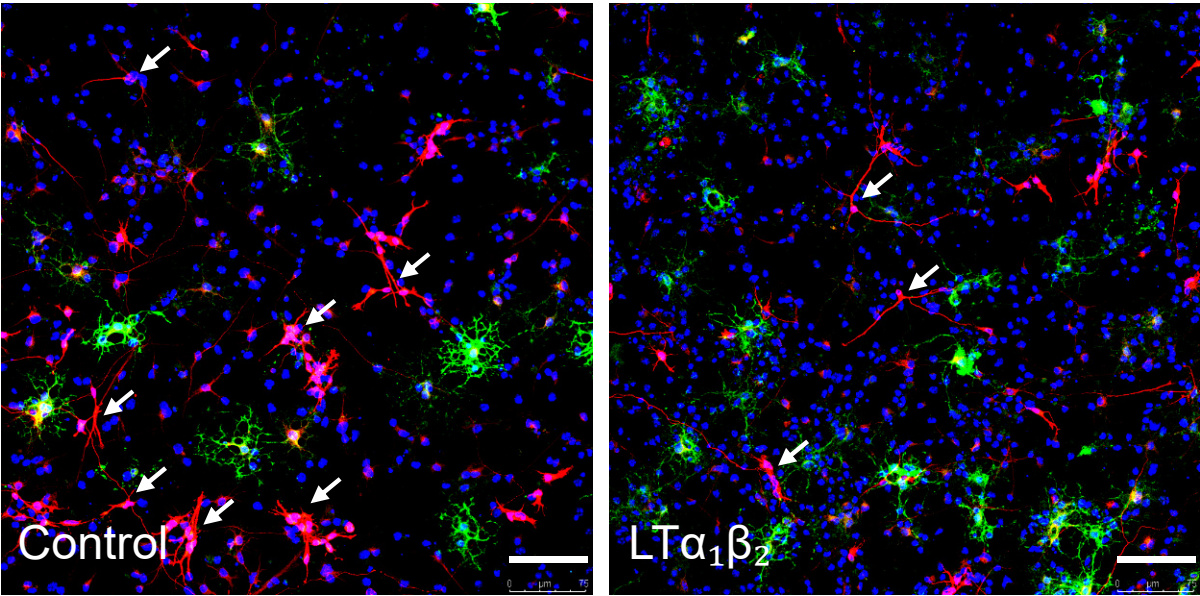

**b** **GFAP/DCX/DAPI**

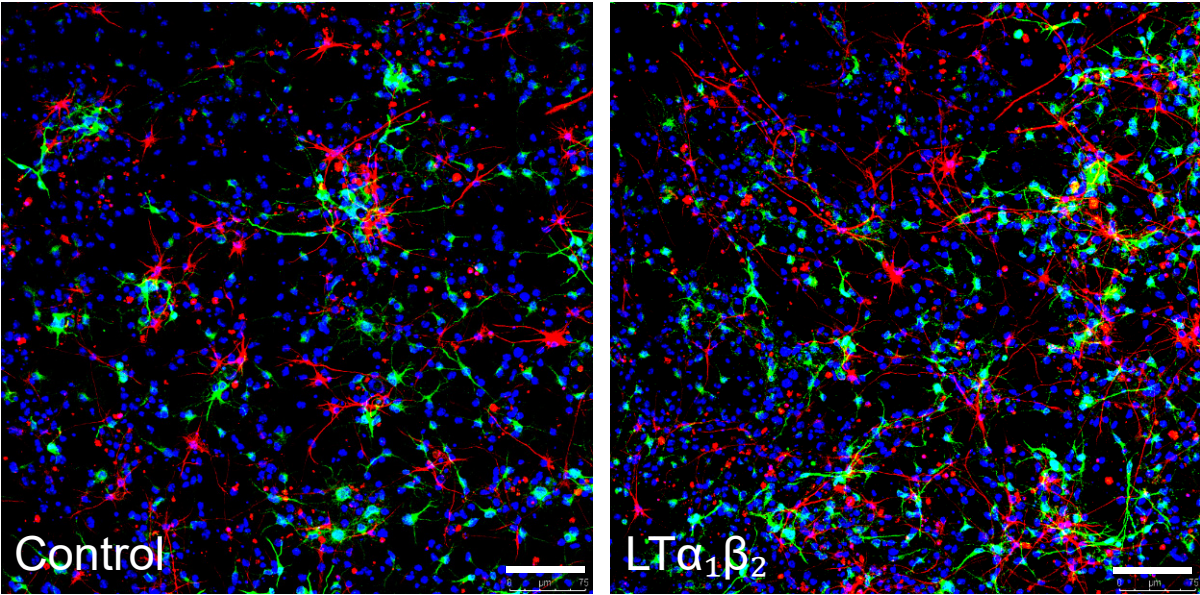

**c**

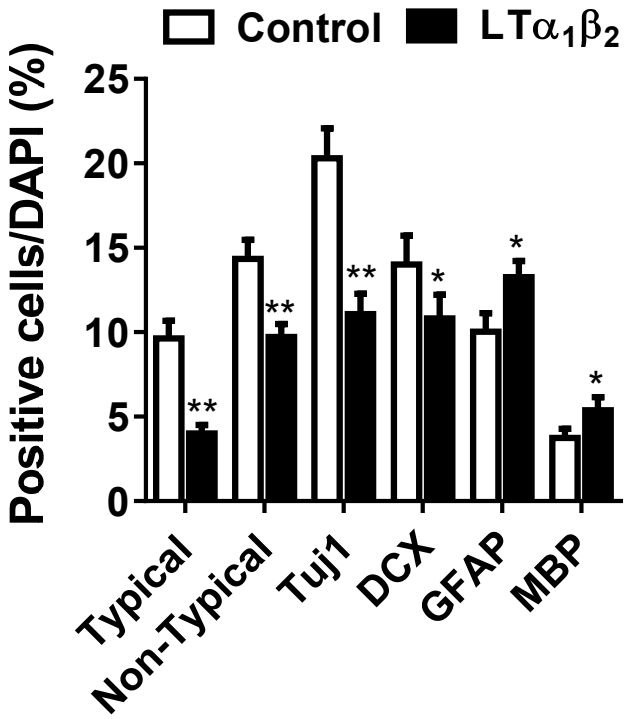

Fig. Sup 3

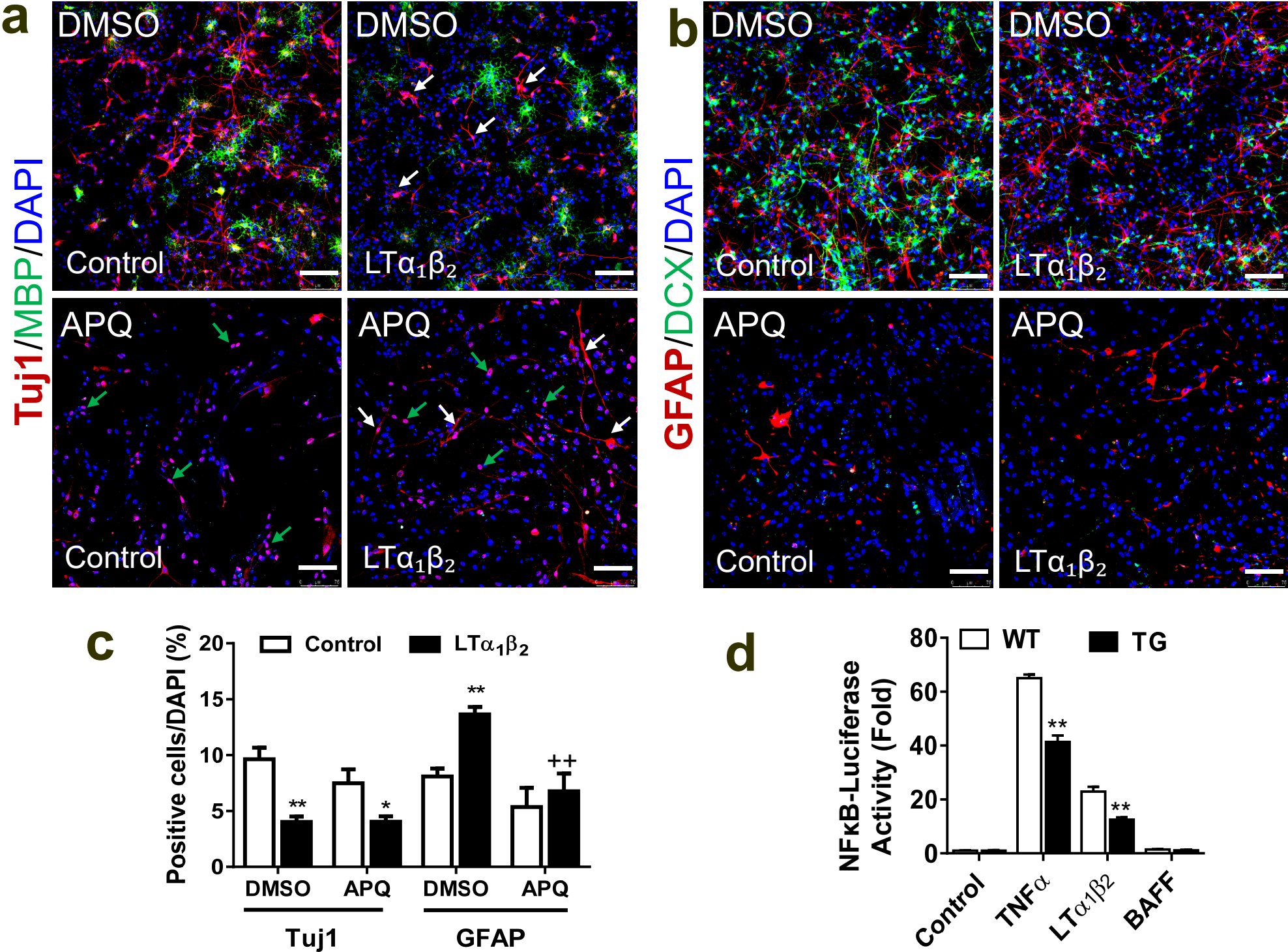

Supplement: Supplementary file 1 — Figure S1. Representative micrographs showing transgenic inactivation of astroglial NFκB pathway inhibits constitutive and LT-induced astroglial lineage differentiation but favors neuronal lineage differentiation in SVZ NSCs/NPCs from littermated wild-type (WT, a) and GFAP-dnIκBα transgenic mice (TG, b). At the 1st, 3rd and 6th day of differentiation, GFAP (green) and DCX (red)-positive cells were determined by multi-labeled fluorescent immunocytochemistry and confocal image analysis. Scale bars= 50 µm. Figure S2. LTα1β2 promoted astrocytic and oligodendrocytic differentiation but inhibited neuronal differentiation in mouse embryonic NSCs/NPCs. (a, b) Representative micrographs showing 3 lineage differentiation of cultured NSCs/NPCs from E14 mouse brain. White arrows (a) indicate representative Tuj1-positive typical neurons with various degrees of neurites and branches. The small red dots in GFAP staining (b) derived from non-specific debris for chicken anti-GFAP antibody. Scale bars = 75 µm. (c) Quantitative analysis of lineage differentiation after treatment with LTα1β2. Figure S3. Effects of NFκB inhibition on neural lineage differentiation in mouse NSCs/NPCs. (a, b) Representative micrographs showing complete loss of DCX (neuroblasts) and MBP (oligodendrocytes) and dramatic reduction of Tuj1 (immature neurons) and GFAP (astrocytes) after NFκB activation inhibitor APQ (10 µM) pretreatment 30 min before LTα1β2 (100 ng/ml) treatment under differentiation condition for 3 days. White arrows indicate representative Tuj1-positive typical neurons and green arrows show the nuclear location of Tuj1 expression (a). Scale bars = 75 µm. (c) Quantitative analysis of Tuj1 and GFAP positive cells after LTα1β2 treatment in the presence or absence of APQ. (d) Adenovirus-mediated NFκB-firefly-luciferase reporter assay showing a significant reduction in cytokine-induced NFκB activation in SVZ NSCs/NPCs from TG mice. Data represent mean ± SEM. * p<0.05 and ** p<0.01 indicate signifi [file 12974_2018_1074_MOESM1_ESM.pdf]
